# On a neural phonon model of EEG brain dynamics

**DOI:** 10.1007/s10827-026-00926-9

**Published:** 2026-03-16

**Authors:** Christopher Batterton, Simeon Ensing, Craig Shepherd, Mahonri Owen, Jemma König, Mitchell Head

**Affiliations:** Te Kura Rau Mahara, Division of STEM, University of Waikato, Hamilton, New Zealand

**Keywords:** Electroencephalography, EEG modelling, Stochastic oscillator networks, Stuart-Landau dynamics, Quantized vibrational modes, Large-scale cortical dynamics, Hamiltonian operator, Neural phonon

## Abstract

Neuronal oscillations are a ubiquitous feature of brain activity, indexing functions from sensory selection to memory formation. Yet a unified framework that (i) accommodates the nonlinear, noise-driven nature of cortical dynamics and (ii) explains standard empirical measures—power, spectral entropy, coherence, Phase-Locking Value (PLV), Phase-Amplitude Coupling (PAC), and envelope correlations—remains elusive. A natural candidate is the noisy Stuart–Landau (SL) oscillator, whose deterministic form models cortical rhythms as limit cycles, while additive noise induces stochastic phase and amplitude fluctuations. Prior work has shown that networks of SL oscillators can replicate burst statistics, multistability, and cross-frequency modulation in electroencephalography/magnetoencephalography (EEG/MEG). However, an analytical framework linking these models directly to observed connectivity metrics has been lacking. Here we derive such a framework by mapping the Fokker–Planck equation (FPE) of each SL oscillator to an imaginary-time Schrödinger operator via a classical similarity transform. A second-order expansion around the limit-cycle amplitude yields a quadratic Hamiltonian whose ladder operators describe quantised fluctuations—neural phonons—in oscillatory power. Bilinear coupling terms inherited from diffusion give rise to analytically diagonalisable bosonic interactions. This construction yields closed-form expressions for spectral observables and their dynamics, including Green-function-derived coherence and PLV, perturbative PAC, and a five-parameter “personality map” linking microscopic physics to macroscopic brain states. By unifying noisy limit-cycle theory with operator methods from statistical physics, we introduce a tractable, interpretable formalism for understanding neural coherence as the dynamics of quantised phonons.

## Introduction

The modelling of brain dynamics through coupled oscillatory systems has a long tradition in computational neuroscience (Buzsáki, 2006; Paul & Bressloff, 2011; Viktor et al., 2013). Recent work has further emphasised the importance of nonequilibrium statistical physics in large-scale brain dynamics (Nartallo-Kaluarachchi et al., 2024). Linear and nonlinear oscillators have been used to approximate EEG and MEG signals across a range of spatiotemporal scales (Breakspear, 2017; Deco et al., 2021). Among these, Stuart–Landau and Hopf-based oscillators are widely used to describe local and network-level rhythmic activity, particularly near bifurcation points where the brain exhibits maximal sensitivity and dynamical richness (Freyer et al., 2012).

Stuart–Landau networks have been used to investigate: whole-brain metastability and turbulence (Deco et al., 2021), phase locking and cluster synchronisation (Kuramoto, 2003), cross-frequency coupling and mode–mode interactions (Adriano et al., 2010; Ryan et al., 2010), resting-state rhythmic variability and transient bursts (Mill et al., 2021). These models typically operate in the mesoscopic regime, where population-level oscillators interact through coupling matrices representing structural or functional connectivity (Breakspear, 2017). However, to date, these approaches have not framed neural oscillations in terms of phonon-like dynamics—that is, as spatially propagating collective vibrational modes governed by coherent energy exchange across a networked medium.

In condensed matter physics, phonons represent quantised modes of molecular lattice vibrations and serve as carriers of energy, coherence, and information (Gyaneshwar & Srivastava, 1990). The mathematical description of phonons, especially in their harmonic limit, aligns closely with the form of nonlinear coupled oscillator models already used in brain modelling. This includes: coupled amplitude–phase dynamics, interactions across space and frequency, mode–mode coupling (analogous to cross-frequency PAC), stochastic excitation and metastable transitions.

Despite these deep conceptual parallels, a phonon-theoretic framing of brain dynamics has not yet been formalised in the computational neuroscience literature. To our knowledge, this work represents the first attempt to model EEG activity explicitly as a **neurophononic field**: a lattice of nonlinear oscillators whose interactions give rise to coherent, frequency-selective vibrational modes, i.e., neural phonons.

We show that each SL oscillator, when mapped through the FPE in polar coordinates and subjected to the classical similarity transform $P = \psi \, e^{-U/2D}$, becomes equivalent to a Schrödinger operator in imaginary time (Risken, 1996). A second-order expansion of the effective potential around the deterministic limit cycle yields a harmonic oscillator whose creation and annihilation operators define quantised amplitude fluctuations, which we refer to as “neural phonons”. The coupling terms inherited from the original SL network appear as bilinear bosonic interactions in a quadratic Hamiltonian, which can be analytically diagonalised.

This formalism leads to closed-form expressions for the relaxation spectrum, autocorrelation times, magnitude-squared coherence, PLV, and PAC, all within a common operator framework. The resulting phonon field description reveals structured amplitude and phase interactions that are inaccessible to uncoupled or single-mode models, and offers a rigorous path for interpreting large-scale brain dynamics using the language of vibrational physics.

A full list of variables, units, and notation used throughout the manuscript is provided in Appendix A (Table 3).

## Stochastic limit cycles, Fokker-Planck probability evolution, and the emergent quantum potential

EEG signals are typically recorded as voltage fluctuations in the microvolt ($\mu \text {V}$) range, representing summed postsynaptic activity across neuronal populations. To extract dynamic features such as amplitude and phase, one common approach is to compute an analytic signal via a complex-valued time-frequency transform (for example, a Morlet wavelet or Hilbert transform). This analytic signal has the form $z(t)=x(t)+i\mathcal {T}[x(t)]=r(t) e^{i \phi (t)}$.

We model neural activity using the Stuart–Landau oscillator, corresponding to the normal-form amplitude equation of a Hopf bifurcation (often referred to as a “Hopf oscillator” in the neuroscience literature). Here we retain a general complex nonlinear coefficient to allow amplitude–phase coupling:

1 $$\begin{aligned} \dfrac{dz}{dt} = (\lambda + i\omega ) z - (\zeta + i\chi ) |z|^2 z + \eta (t) \end{aligned}$$

where $z(t) \in \mathbb {C}$ is the oscillator state trajectory on the complex plane $\mathbb {C}$, the parameter $\lambda$ governs linear growth, $\zeta$ sets the nonlinear saturation, $\omega$ is the intrinsic frequency, and $\chi$ determines the strength of amplitude–phase coupling. The additive noise $\eta (t) \in \mathbb {C}$ is a complex-valued white noise process modeled as a delta-correlated Gaussian noise process, with positive real-valued noise intensity $D\in \mathbb {R}^+$ and autocorrelation $\langle \eta (t) \eta ^*(t') \rangle = 2D\, \delta (t - t')$. Transforming into polar coordinates $z = r e^{i\phi }$, we separate the signal dynamics into radial (*r*) and angular ($\phi$) components:

2 $$\begin{aligned} \dfrac{dr}{dt}&= \lambda r - \zeta r^3 + \Re \{e^{-i\phi } \eta (t)\} = f_r(r) + \eta _r(t) \end{aligned}$$

3 $$\begin{aligned} \dfrac{d\phi }{dt}&= \omega - \chi r^2 + \frac{1}{r} \Im \{e^{-i\phi } \eta (t)\} = f_\phi (r) + \eta _\phi (t) \end{aligned}$$

where $f_r(r) = \lambda r - \zeta r^3$ and $f_\phi (r) = \omega - \chi r^2$ are ‘forces’ governing the deterministic evolution of the signal’s amplitude and phase, respectively. The stochastic terms $\eta _r$ and $\eta _\phi$ inject noise in the signal’s amplitude and phase evolutions as modulated by *D* via the real ($\Re$) and imaginary ($\Im$) components of the SL noise, $\eta$.

The SL model can be solved exactly in the absence of noise ($D=0$) to yield the deterministic trajectories of amplitude and phase

4a $$\begin{aligned} r(t)&= r_e \left( 1 + \left( \dfrac{r_0^2}{r_e^2} - 1 \right) e^{-2\lambda t} \right) ^{-1/2}\end{aligned}$$

4b $$\begin{aligned} \phi (t)&= \phi _0 + \omega t - \dfrac{\chi }{2\zeta } ~ \ln \left( 1 + \dfrac{r_0^2}{r_e^2} (e^{2\lambda t} -1 ) \right) \end{aligned}$$

for initial state $z_0=r_0e^{i\phi _0}$ with equilibrium amplitude $r_e=\sqrt{\lambda /\zeta }$. The amplitude monotonically plateaus to its equilibrium from any initial value with a rate due to $\lambda$. The instantaneous frequency $\frac{d\phi }{dt}$ therefore also monotonically plateaus to a value of $f_\phi (r_e)=\omega -\chi r_e^2=\omega -\chi \lambda /\zeta$, showing the transient nature of the phase-amplitude coupling. The deterministic SL oscillator will quiesce into a limit cycle of a constant amplitude and frequency.

**Fig. 1:**
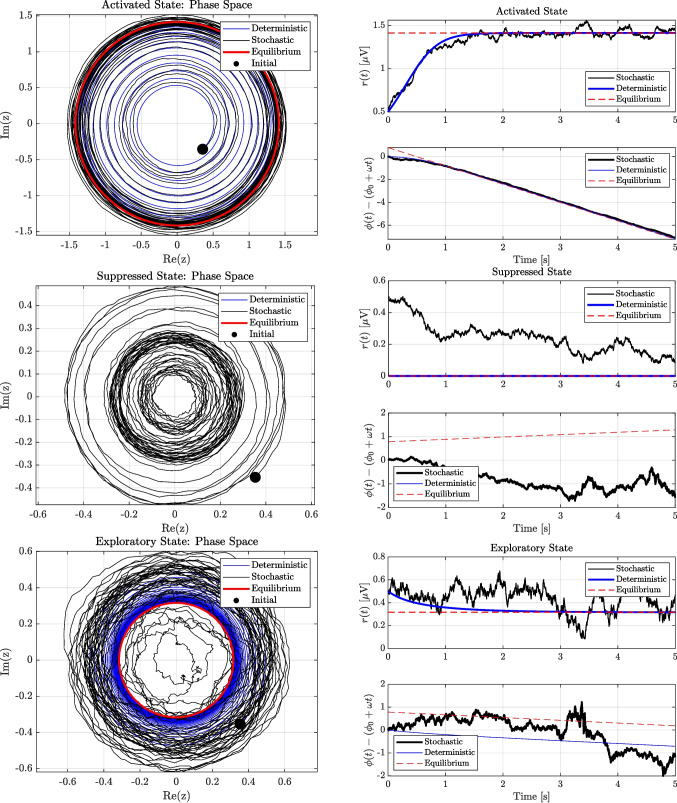
Numerical simulations of an alpha-band EEG signal (10 Hz) via the stochastic Stuart–Landau (SL) oscillator under three archetypal regimes: activated, suppressed, and exploratory. Phase space trajectories (*left*) and corresponding time series of amplitude and phase drift (*right*) are shown. Simulated stochastic trajectories (black) are compared with the deterministic solution (blue) and equilibrium amplitude $r^e = \sqrt{\lambda /\zeta }$ (red). Parameters for each regime are detailed Table 1

Simulations of the noisy SL model under activated, suppressed and exploratory archetypal cognitive states are shown in Fig. 1.

The *activated* regime represents sustained oscillatory activity around a stable limit cycle, with high signal coherence and low noise. The *suppressed* regime lacks a limit cycle due to negative linear growth, resulting in quiescent, noise-dominated dynamics. The *exploratory* regime exhibits broadband, high-entropy activity, combining moderate excitation with high damping and noise. These regimes are instantiated via distinct combinations of model parameters as summarised in Table 1.

**Table 1:** Parameter values used to simulate different cognitive regimes of the SL oscillator

Regime	$\lambda$ [s$^{-1}$]	$\zeta$ [$\mu$V$^{-2}$s$^{-1}$]	$\chi$ [$\mu$V$^{-2}$s$^{-1}$]	*D* [$\mu$V$^2$s$^{-1}$]
Activated	2.0	1.0	0.8	0.010
Suppressed	$-0.5$	1.0	0.2	0.005
Exploratory	0.5	5.0	1.2	0.050

Under the Stratonovich interpretation, these equations lead to a two-dimensional Fokker–Planck equation (FPE) for the joint probability density $P(r, \phi , t)$ (Risken, 1996). We now focus on the radial component, integrating over $\phi$ to obtain the marginal density $P(r, t)$. Using $f_r$ as a radial drift function, the radial FPE becomes

5 $$\begin{aligned} \frac{\partial P}{\partial t} = -\frac{\partial }{\partial r} \left[ f_r(r) P \right] + D \left[ \frac{\partial ^2 P}{\partial r^2} + \frac{1}{r} \frac{\partial P}{\partial r} - \frac{P}{r^2} \right] \end{aligned}$$

which tracks the deterministic drift and stochastic diffusive behaviours of the oscillator. We now perform a similarity transformation $P(r, t) = \psi (r, t) e^{-U(r)/2D}$, with effective potential

6 $$\begin{aligned} U(r) = -\int f_r(r) \, dr = - \frac{1}{2} \lambda r^2 + \frac{1}{4} \zeta r^4 \end{aligned}$$

The stationary probability density due to this deterministic drift potential has a Boltzmann distribution, $P_s(r)~=~\mathcal {N}^{-1}\exp (-U(r)/D)$, for normalisation constant $\mathcal {N}$. In the long-time limit, the Shannon entropy is due to the average (first moment) of *U* and noise intensity

7 $$\begin{aligned} S(r) = - \int dr ~ P_s(r) \ln P_s(r) ~=~ \dfrac{\langle U (r) \rangle }{D} + \ln \mathcal {N} \end{aligned}$$

Substituting into the FPE and simplifying, we obtain an imaginary-time Schrödinger equation, $-\hbar \partial _t\psi = \hat{\mathcal {H}}\psi$, for wavefunction $\psi (r, t)$:

8 $$\begin{aligned} -\frac{\partial \psi }{\partial t} = \left[ -D \frac{\partial ^2}{\partial r^2} + V(r) \right] \psi \end{aligned}$$

Equating the first term to the radial kinetic operator, $\frac{\hbar ^2}{2m} \partial _r^2$, demonstrates the well-known equivalence $D=\hbar /2m$ for an effective mass *m* (Risken, 1996). In the current context, $\hbar$ is a fluctuation localisation scale and governs the spatial scale over which probability densities spread, with a larger value indicating greater delocalisation of amplitude fluctuations about the limit cycle.

At this stage, we retain the freedom to choose $\hbar$ and *m* independently, provided that they satisfy the kinetic correspondence. To maintain dimensional and conceptual clarity, we fix $\hbar$ to have units of “EEG action”, specifically, $\mu \text {V}.s$. This choice casts $\hbar$ as a localisation metric: it governs the spatial concentration of probability mass in the transformed wavefunction $\psi$, much like in quantum mechanics where smaller $\hbar$ leads to tighter localisation of the wavefunction around classical paths. Accordingly, the effective mass *m* inherits the dimension of time, representing a temporal stiffness or memory depth — how long a mode resists stochastic broadening. This interpretation links the statistical mechanics of neural fields to canonical quantum behaviour while remaining grounded in electrophysiological observables.

**Fig. 2:**
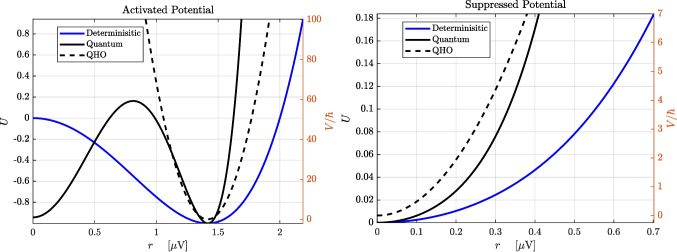
Potential landscapes derived from the SL oscillator’s effective potential $V(r)$. Left: Activated state with limit cycle, $r_e=\sqrt{2}~\mu$V. Right: Suppressed state with no limit cycle, $r_e=0~\mu$V. Black curves: full quantum potential; blue: deterministic drift potential $U(r)$; dashed: quadratic harmonic approximation. Minima correspond to equilibrium amplitudes used in Fig. 1

The second term is an effective potential given by

9 $$\begin{aligned} V(r) = \dfrac{\hbar }{2} \left( \dfrac{df_r}{dr} ~+~ \dfrac{f_r^2}{2D} \right) = \dfrac{\hbar }{2} \left( \lambda - 3 \zeta r^2 + \dfrac{1}{2D} (\lambda - \zeta r^2)^2 r^2 \right) \end{aligned}$$

which is a sixth-order polynomial that is symmetric about $r=0$ which allows for the existence of tri-stability regimes for certain ranges of the parameter space. This equation defines stochastic quantum-like dynamics for the radial component, with $D$ playing the role of an effective mass *m *and Planck constant $\hbar$, and $V(r)$ acting as a confining potential whose curvature determines the amplitude stiffness (Haken, 1983). The potential energy landscapes for the activated and suppressed cognitive states are shown in Fig. 2.

We now extend the Fokker–Planck–to–Schrödinger mapping to include the full joint probability density $P(r, \phi , t)$, which captures both amplitude and phase dynamics. Starting from the polar-form SL Eq. (3), the associated FPE reads:

10 $$\begin{aligned} \frac{\partial P}{\partial t} = - \frac{\partial }{\partial r} [f_r(r) P] - \frac{1}{r} \frac{\partial }{\partial \phi } [f_\phi (r) P] + D \left[ \frac{\partial ^2 P}{\partial r^2} + \frac{1}{r} \frac{\partial P}{\partial r} + \frac{1}{r^2} \frac{\partial ^2 P}{\partial \phi ^2} \right] \end{aligned}$$

We now apply a similarity transform:

11 $$\begin{aligned} P(r, \phi , t) = \psi (r, \phi , t) \exp \left( -\frac{U(r)}{2D} \right) , \end{aligned}$$

where $$ U(r)$$ is the radial drift potential Eq. (6). This transforms the FPE into a Schrödinger-like equation in imaginary time:

12 $$\begin{aligned} - \frac{\partial \psi }{\partial t} = \left[ - D \left( \frac{\partial ^2}{\partial r^2} + \frac{1}{r} \frac{\partial }{\partial r} + \frac{1}{r^2} \frac{\partial ^2}{\partial \phi ^2} \right) + V(r) - i f_\phi (r) \frac{\partial }{\partial \phi } \right] \psi \end{aligned}$$

where the first term is the polar kinetic energy $\frac{\hbar ^2}{2m} \nabla ^2$, and the term $-i f_\phi \partial _\phi$ describes angular drift analogous to a synthetic vector potential (Aharonov & Bohm, 1959; Haken, 1983).

The final operator has the structure of a two-dimensional Hamiltonian on a disc, with a nonlinear radial potential, Laplace–Beltrami kinetic terms, and a phase-drift term that plays the role of a background gauge field. This interpretation allows us to map amplitude–phase coupling ($\chi$) to a synthetic magnetic flux and to derive closed-form expressions for observables such as PAC and PLV using perturbation theory and Green’s functions (Adriano et al., 2010; Breakspear, 2017). In the next section, we will quantise this operator and introduce a global Hamiltonian governing the coupled dynamics of multiple SL oscillators.

## Neural phonon Hamiltonian with amplitude and phase coupling

To describe the cortex’s global amplitude dynamics, we begin with the amplitude-only part of the stochastic Stuart–Landau network with *N* electrodes subject to coupling $\sum _{k=1}^N K_{jk}\cdot (z_k-z_j)$, for oscillators at electrode sites $j$ and $k$. We write the amplitude interaction for the oscillator at electrode channel site *j* with the other channels *k* as

13 $$\begin{aligned} \dfrac{dr_j}{dt} = f_r(r_j) + \eta _{r_j}(t) + \sum _{k=1}^{N} \Re ( K_{jk} ) \cdot (r_k - r_j) \end{aligned}$$

where $f_r(r_j)$ describes the local nonlinear dynamics, and $\Re (K_{jk})$ denotes the real part of the complex coupling matrix, which governs diffusive amplitude exchange throughout the network. The corresponding effective potential for amplitude fluctuations is then

14 $$\begin{aligned} \mathcal {H}_{\text {eff}} = \sum _{j=1}^{N} V(r_j) + \frac{1}{2} \sum _{j \ne k}^N S_{jk} (r_j - r_k)^2 \end{aligned}$$

where assuming a bidirectional equivalence in oscillator coupling, $K_{kj}=K_{jk}$, permits a symmetric coupling matrix $S_{jk} = -\Re (K_{jk})$. Expanding this expression to second order around the deterministic limit-cycle amplitudes $r_j^e =\sqrt{\lambda _j/\zeta _j}$, we obtain a quantum harmonic oscillator approximation:

15 $$\begin{aligned} \mathcal {H}_{\text {qho}} = \frac{1}{2} \sum _{j=1}^{N} V''(r_j^e) \delta r_j^2 + \frac{1}{2} \sum _{j \ne k}^N S_{jk} (\delta r_j - \delta r_k)^2 \end{aligned}$$

where $\delta r_j = r_j - r_j^e$ and $V''(r_j^e) =2\hbar \lambda _j^2/D_j - 3\hbar \zeta _j$ denotes the curvature of the local effective potential well minima. From this expansion, the dynamical matrix $\mathbf {\Phi } =\textbf{M}^{-1/2} \textbf{H} \textbf{M}^{-1/2}$, for a local curvature (Hessian evaluated at equilibrium) $[\textbf{H}]_{jk}=\partial _{r_j}\partial _{r_k}\hat{\mathcal {H}}_\text {qho}|_\text {min}$, is assembled with elements

16 $$\begin{aligned} \Phi _{jj}&= \frac{1}{m_j} \left( V''(r_j^e) + \sum _{k \ne j}^N S_{jk} \right) \end{aligned}$$

17 $$\begin{aligned} \Phi _{jk}&= -\frac{S_{jk}}{\sqrt{m_j m_k}}, \quad j \ne k \end{aligned}$$

where $m_j = \hbar / 2D_j$ is the effective mass derived from noise level $D_j$. This structure shows that: diagonal terms arise from local curvature of the potential $V_{eff}$, and off-diagonal terms reflect diffusive coupling between oscillators. Diagonalising $\mathbf {\Phi }$ yields normal modes $\vec {v}^{(\ell )}$ and eigenfrequencies $\Omega _\ell$ via the eigenequation

18 $$\begin{aligned} \mathbf {\Phi } \vec {v}^{(\ell )} = \Omega _\ell ^2 \vec {v}^{(\ell )} \end{aligned}$$

analogous to vibrational normal mode analysis in molecules where the Hessian matrix governs coupled atomic motion (Gyaneshwar & Srivastava, 1990). This serves as a change of basis from individual oscillators (electrode channel sites) to a global vibrational mode index of neural phonons spanning the cortex, in much the same way as how the molecular dynamical matrix (mass-weighted Hessian of equilibrium potential) maps the perturbations of bound atomic nuclei in 3D space to a summation of molecular vibrational modes.

The eigenvector element $v_j^{(\ell )}$ is proportional to the fluctuation amplitude at electrode *j* due to mode $\ell$; the corresponding element in molecular physics encodes the perturbation of an atomic nucleus from its equilibrium position (polarisation) due to a vibrational mode. The eigenvector columns, $\vec {v}^{(\ell )}$ therefore describe the spatial patterning of the neural phonon that oscillates with an eigenfrequency $\Omega _\ell$.

In this framework, the number of vibrational modes is equal to the number of electrodes which sample the local oscillators (*N*). Each mode forms part of a complete orthonormal basis for the amplitude field, and the full set of $\{\vec {v}^{(\ell )}\}$ spans all admissible cortical amplitude fluctuations. The ensures that the EEG voltage dynamics can be fully reconstructed from the linear superposition of *N* quantised neural phonons.

It is important to distinguish the intrinsic oscillator frequency $\omega _j$, which appears in the SL model, and the collective phonon-mode frequency $\Omega _\ell$, which emerges from diagonalising the dynamical matrix. The quantity $\omega _j$ determines the natural phase rotation rate of the uncoupled SL oscillator *j*, reflecting its intrinsic rhythmic tendency in the absence of interactions. In contrast, $\Omega _\ell$ is the eigenfrequency of vibrational mode $\ell$, shaped by both the local curvature of the effective potential and the network-wide coupling structure. While both quantities have units of temporal frequency, they both describe fundamentally different dynamical properties: $\omega _j$ is local and phase-based, whereas $\Omega _\ell$ is global and amplitude based.

The phonon-mode mass $M_\ell$ is derived from the distribution of local oscillator noise effective masses $m_j=\hbar /2D_j$ and the spatial structure of eigenmode $\vec {v}^{(\ell )}$. Specifically,

19 $$\begin{aligned} M_\ell = \sum _{j=1}^N m_j \left( v_j^{(\ell )} \right) ^2 \end{aligned}$$

which expresses the collective inertia of mode $\ell$ as a weighted sum over local temporal diffusion resistances. This parallels the molecular vibrational analysis where (normal mode) phonon masses are constructed from mass- and polarisation-weighted contributions of each atom in the molecular undulation. In the neural case, $m_j$ reflects the local noise level at site *j*, and the global phonon mass $M_\ell$ incorporates both spatial and stochastic structure.

For an isolated mode in the absence of coupling, the corresponding eigenfrequency is given by a curvature-weighted average:

20 $$\begin{aligned} \Omega _\ell ^2 = \dfrac{1}{M_\ell } \sum _{j=1}^N V''(r_j^{e}) \left( v_j^{(\ell )} \right) ^2 \end{aligned}$$

which reflects how the local potential curvature contributes to global mode stiffness, filtered through the spatial mode structure and noise-dependent local effective masses.

To describe the vibrational energy of the cortical amplitude field, we begin with the quantised Hamiltonian for a set of decoupled harmonic phonon modes, each defined by conjugate operators $\hat{q}_\ell$ and $\hat{p}_\ell$ in the standard form (Gyaneshwar & Srivastava, 1990)

21 $$\begin{aligned} \mathcal {H}_\text {amplitude} = \sum _{\ell =1}^{N} \left( \frac{1}{2 M_\ell } \hat{p}_\ell ^2 + \frac{1}{2} M_\ell \Omega _\ell ^2 \hat{q}_\ell ^2 \right) \end{aligned}$$

This allows $\hat{q}_\ell$ to be interpreted as a phonon-induced harmonic amplitude fluctuation about the limit cycle $r_j^e$, with conjugate phonon momenta $\hat{p}_\ell$. We quantise the radial mode dynamics using bosonic ladder operators via standard second quantisation:

22a $$\begin{aligned} \hat{a}_\ell&= \sqrt{\dfrac{M_\ell \Omega _\ell }{2\hbar }} ~ \left( \hat{q}_\ell + \dfrac{i}{M_\ell \Omega _\ell } \hat{p}_\ell \right) \end{aligned}$$

22b $$\begin{aligned} \hat{a}_\ell ^\dagger&= \sqrt{\dfrac{M_\ell \Omega _\ell }{2\hbar }} ~ \left( \hat{q}_\ell - \dfrac{i}{M_\ell \Omega _\ell } \hat{p}_\ell \right) \end{aligned}$$

satisfying the canonical commutation relation $[\hat{a}_\ell , \hat{a}_m^\dagger ] = \delta _{\ell m}$. The operator $\hat{a}_\ell$ ($\hat{a}_\ell ^\dagger$) annihilates (creates) a phonon in neural vibrational mode $\ell$, and the operator product $\hat{n}=\hat{a}_\ell ^\dagger \hat{a}_\ell$ gives the number of phonons in mode $\ell$. In terms of the neural ladder operators, the amplitude Hamiltonian becomes:

23 $$\begin{aligned} \mathcal {H}_\text {amplitude} = \sum _{\ell =1}^{N} \hbar \Omega _\ell \left( \hat{n}_\ell + \frac{1}{2} \right) \end{aligned}$$

The expectation value for the number of phonons in neural vibrational mode $\ell$ is the trace of the occupation number operator $\langle \hat{n}_\ell \rangle = \langle \hat{a}_\ell ^\dagger \hat{a}_\ell \rangle$. The expectation excitation energy of the cortex due to the superposition of all neural phonons is then $E = \langle \hat{\mathcal {H}}_r\rangle = \sum _{\ell =1}^{N} \hbar \Omega _\ell \left( \langle \hat{n}_\ell \rangle + \frac{1}{2} \right)$.

This expression is directly analogous to the internal energy of a crystalline solid in the quantum harmonic approximation (Gyaneshwar & Srivastava, 1990). If all $\Omega _\ell$ are equal, the model reduces to an Einstein solid; for a spectrum of mode frequencies, it parallels the full phonon description used in the Debye model of solids, where each vibrational mode contributes quantised energy based on its phonon occupation. Here, the neural phonons play the role of quantised fluctuations, and their superposition determines the total energy in the cortical amplitude field.

From an EEG perspective, this energy spectrum can be interpreted as a structured decomposition of the signal’s power across latent vibrational modes. Rather than viewing EEG power as arising from isolated rhythms, this formalisation frames it as a superposition of quantised fluctuations constrained by the brain’s geometry, coupling, and noise. As in solid state physics, the mode distribution and phonon occupation reveal not just the energy landscape but also the system’s functional state—here, reflecting cognitive state, neuronal arousal, or network coherence.

Finally, we map the phonon displacement modes back into the EEG amplitude space. The amplitude fluctuations about the limit cycle are expressed in terms of the neural phonon annihilation and creation operators by

24 $$\begin{aligned} \hat{q}_\ell = \sqrt{\dfrac{\hbar }{2M_\ell \Omega _\ell } } ~ \left( \hat{a}_\ell ^\dagger + \hat{a}_\ell \right) \end{aligned}$$

The expectation amplitude fluctuation of the EEG signal at electrode *j* is given by the mode superposition

25 $$\begin{aligned} r_j(t) = r_j^e + \sum _{\ell =1}^N \dfrac{v_j^{(\ell )}}{\sqrt{m_j}} ~ \langle \hat{q}_\ell (t) \rangle \end{aligned}$$

This relation shows how global (e.g., cortical) vibrational modes structure the local amplitude field, thereby determining the observable EEG signal at each electrode. In parallel with the quantum harmonic oscillator description of molecules, this construction demonstrates that each neural phonon mode corresponds to a quantised excitation of the cortical amplitude structure, governed by the joint properties of local diffusivity and network-wide coupling geometry.

This completes the explicit construction of the neural phonon spectrum, revealing its structure as a quantised vibrational field shaped by local dynamics and global coupling.

We now assemble the full neural Hamiltonian, combining the quantised amplitude modes with angular phase dynamics and network interactions. Each Stuart–Landau oscillator contributes both a radial (amplitude) and angular (phase) degree of freedom, leading to a composite field description (Breakspear, 2017; Freyer et al., 2012).

Following the quantisation of the radial fluctuations from the previous section, each phonon mode $q_\ell$ evolves according to the harmonic Hamiltonian Eq. (23). Each oscillator also supports a phase degree of freedom with conjugate phase momentum $\hat{p}_{\phi _j} = -i\hbar \, \partial _{\phi _j}$, giving rise to

26 $$\begin{aligned} \mathcal {H}_{\text {phase}} = \sum _{j=1}^{N} \left( \frac{1}{2 I_j} \hat{p}_{\phi _j}^2 - f_\phi (r_j) \hat{p}_{\phi _j} \right) , \end{aligned}$$

where $I_j = m_j r_j^2 = \hbar r_j^2 / 2D_j$ is the effective moment of inertia and $f_\phi (r_j) = \omega _j - \chi _j r_j^2$ determines the amplitude-modulated phase velocity (Haken, 1983). This is equivalent to a charged particle confined to a ring that experiences a vector potential (for example, a magnetic flux) $I_j f_\phi (r_j)$. In quantum mechanics, it is known that a stronger angular drift leads to entrainment, phase locking, asymmetric phase distributions, and shifts energy levels (Aharonov–Bohm effect) (David et al., 2018).

Inter-oscillator phase coupling is captured by cosine terms derived from the imaginary part of the complex SL coupling:

27 $$\begin{aligned} \mathcal {H}_{\text {coupling}} = - \sum _{j<k} J_{jk}(r_j, r_k) \cos (\phi _j - \phi _k), \end{aligned}$$

where the symmetric phase coupling matrix $J_{jk}=-\frac{1}{2} r_j r_k \Im ( K_{jk} )$ represents the effective phase synchronisation strength and depends on the instantaneous amplitudes modulated by the imaginary components of the coupling matrix $K_{jk}$. This structure parallels the coupling used in Josephson junction arrays of superconductors and classical spin models (Barone & Paternò, 1982).

Combining all terms, the total neural Hamiltonian reads

28 $$\begin{aligned} \mathcal {H}_{\text {neural}} = \sum _{\ell =1}^{N} \hbar \Omega _\ell \left( \hat{n}_\ell + \frac{1}{2} \right) \\+ \sum _{j=1}^{N} \left( \frac{1}{2 I_j} \hat{p}_{\phi _j}^2 - f_\phi (r_j) \hat{p}_{\phi _j} \right)\\ - \sum _{j < k}^N J_{jk}(r_j, r_k) \cos (\phi _j - \phi _k). \end{aligned}$$

whose expectation value gives the total energy of the neural field: $E_\text {neural}=\langle \hat{\mathcal {H}}_\text {neural} \rangle$. The three terms correspond to amplitude, phase, and coupling contributions, respectively. The first term encodes the spectral distribution of quantised amplitude fluctuations across the neural phonon modes. The second captures rotational kinetic energy and amplitude-modulated drift in the phase field. The third reflects the energy stored in the inter-oscillator synchrony, with higher values penalising phase desynchronisation.

This expression unifies amplitude phonons, phase angular momentum, and diffusive coupling in a single operator framework. Structurally, it parallels Hamiltonians used in condensed matter physics, including superconducting Josephson arrays, exciton condensates, and phase-locked oscillator lattices. Such analogies open the door to applying powerful tools from spectral theory, perturbation analysis, and topological classification.

Taken together, this formalism provides a thermodynamic basis for interpreting EEG observables: mode energy aligns with spectral power, phase coherence with PLV, and amplitude-phase interactions with PAC. In the next section, we exploit this structure to derive analytic expressions for these quantities and predict how they shift with neural state and model parameters.

## Analytic EEG observables from phonon dynamics

**Fig. 3:**
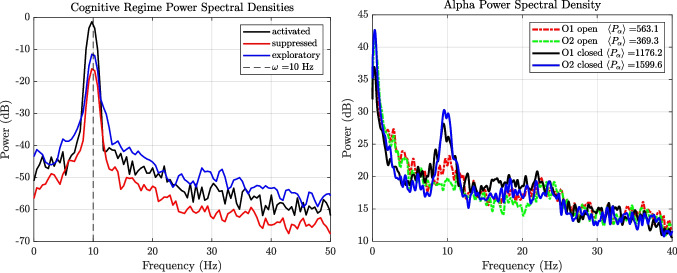
(**a**) Power spectral densities for a simulated EEG signal generated by the SL oscillator in distinct dynamical regimes, illustrating a finite-linewidth Lorentzian peak associated with an activated nonequilibrium mode. The spectra are centered around the intrinsic oscillation frequency $\omega /2\pi = 10$ Hz (dashed line), with broadened Lorentzian peaks whose shape and width reflect the underlying noise strength and damping parameters. These profiles are consistent with the analytical form derived in Eq. (35). (**b**) Empirical EEG recordings of power spectral densities at occipital electrodes (O1, O2) during eyes-open and eyes-closed conditions. Eyes-closed recordings exhibit enhanced and spectrally localised alpha-band power relative to eyes-open recordings, consistent with activation of a dominant collective mode. In addition to this peak, the EEG spectra display a broadband low-frequency 1/*f*-like background that is not present in the isolated oscillator simulation, reflecting ensemble-scale and non-coherent neural activity. Mean alpha band power $\langle P_\alpha \rangle$ is indicated for each condition

To provide emperical support for the model, we draw a comparison of the activated, suppressed and exploratory regimes of the SL model with EEG data shown in Fig. 3. The empirical EEG spectra in Fig. 3 arise from real resting-state recordings acquired in our laboratory from a healthy adult participant (author M.H.) using a 14-channel Emotiv EPOC-X headset during a simple eyes-open / eyes-closed protocol. These measurements provided direct experimental motivation for the present neural phonon formulation and illustrate how the analytic observables derived here connect naturally to measurable cortical spectral dynamics.

The SL model Eq. (1) describes an individual signal at a well-defined frequency. We now extend the model to a multiband description for each channel to attempt to describe the cortical spectral dynamics as measured from an array of EEG scalp electrodes.

For each EEG channel $j$, we model the local complex-valued time-series signal $z_j^{(b)}(t) \in \mathbb {C}$, and partition the spectral behaviour into the conventional activity bandwidths $b \in \{{ delta},\,{ theta},\,{ alpha},\,{ beta},\,{ gamma}\}$. The signal $z_j^{(b)}$ may be interpreted either as the analytic signal extracted from experimental EEG using a Morlet wavelet or Hilbert transform at band $b$, or the solution of the multiband SL oscillator model. This dual role of $z_j^{(b)}$ allows direct comparisons between simulated and real EEG dynamics, and enables model parameters to be constrained from empirical data.

We now define several standard EEG observables directly in terms of the complex-valued analytic signal $z_j^{(b)}(t)$, where $j$ indexes the channel and $b$ the frequency band. The power spectral density for an analytic signal can be computed using Welch’s method, for example.

The Amplitude–Amplitude Coupling (AAC) is defined using the instantaneous amplitude of each bandpass component $|z_j^{(b)}(t)|$. The AAC between two signals is given by Palva and Palva (2007):

29 $$\begin{aligned} \text {AAC}_{jk}^{(b,b')}(t) = |z_j^{(b)}(t)| \cdot |z_k^{(b')}(t)| \end{aligned}$$

Statistical association (e.g., correlation or mutual information) between these amplitude envelopes quantifies their long-range co-modulation. PAC captures how the amplitude of a faster rhythm is modulated by the phase of a slower one (Canolty et al., 2006). It is given by:

30 $$\begin{aligned} \text {PAC}_j^{(b,b')}(t) = |z_j^{(b')}(t)| \cdot \cos [\arg z_j^{(b)}(t)] \end{aligned}$$

where band $b$ is lower (phase source), and band $b'$ is higher (amplitude target). A variety of summary statistics (e.g., modulation index) can be computed from this time series. The PLV measures the synchrony of instantaneous phase between two signals:

31 $$\begin{aligned} \text {PLV}_{jk}^{(b)} = \left| \left\langle e^{i \Delta \phi _{jk}^{(b)} } \right\rangle _t \right| = \left| \left\langle \exp \left[ i \left( \arg z_j^{(b)}(t) - \arg z_k^{(b)}(t) \right) \right] \right\rangle _t \right| \end{aligned}$$

where $|\langle \cdot \rangle _t|$ represents the absolute value of the signal’s mean average in time. It reflects the temporal stability of the phase difference and ranges from 0 (no locking) to 1 (perfect synchrony) (Lachaux et al., 1999). Coherence between channels $i$ and $j$ in band $b$ is defined as:

32 $$\begin{aligned} \rho _{ij}^{(b)}(t) = \frac{|\langle z_i^{(b)} z_j^{(b)*} \rangle |}{\sqrt{\langle |z_i^{(b)}|^2 \rangle \langle |z_j^{(b)}|^2 \rangle }} \end{aligned}$$

which is then normalised across all pairs, $\tilde{\rho }_{ij}^{(b)}(t) = \rho _{ij}^{(b)}(t)/\sum _{i,j} \rho _{ij}^{(b)}(t)$, leading to the coherence entropy (King et al., 2013):

33 $$\begin{aligned} H_\rho ^{(b)}(t) = -\sum _{i,j} \tilde{\rho }_{ij}^{(b)}(t) \log \tilde{\rho }_{ij}^{(b)}(t) \end{aligned}$$

Lower $H_\rho$ indicates strong phase-based network synchrony; higher values indicate desynchronisation or spectral dispersion.

We now use the full phonon-based Hamiltonian to derive closed-form expressions for EEG observables, using a combination of Green’s function analysis and perturbative corrections (Breakspear, 2017; Risken, 1996).

Let each phonon mode $\ell$ evolve as an undamped driven harmonic oscillator with an expectation amplitude fluctuation $q_\ell =\langle \hat{q}_\ell \rangle$ via the equation of motion, $\ddot{q}_\ell + \Omega _\ell ^2 q_\ell = \tilde{\eta }_\ell (t)$, where $\tilde{\eta }_\ell (t)$ is a noise term projected into the eigenbasis. Taking the Fourier transform yields

34 $$\begin{aligned} \tilde{q}_\ell (\omega ) = \frac{\tilde{\eta }_\ell (\omega )}{\Omega _\ell ^2 - \omega ^2} \end{aligned}$$

so the Green’s function is $G_\ell (\omega ) = (\Omega _\ell ^2 - \omega ^2)^{-1}$, and the power spectral density becomes

35 $$\begin{aligned} S_{q_\ell }(\omega ) = |G_\ell (\omega )|^2 S_{\eta _\ell }(\omega ) = \frac{2\hbar }{(\Omega _\ell ^2 - \omega ^2)^2} \end{aligned}$$

This expression yields Lorentzian spectral peaks that can be directly compared to EEG data (Deco et al., 2021; Mill et al., 2021). We note that these works do not claim Lorentzian forms for whole-EEG power spectra, but rather motivate the use of low-dimensional oscillator-based and nonequilibrium modelling approaches to describe collective neural dynamics.

The EEG signal at site $j$ due to all modes is Eq. (25) so the cross-spectrum is

36 $$\begin{aligned} S_{jk}(\omega ) = \sum _{\ell =1}^{N_v} \frac{v_j^{(\ell )} v_k^{(\ell )}}{\sqrt{M_j M_k}} S_{q_\ell }(\omega ) \end{aligned}$$

and coherence is computed as

37 $$\begin{aligned} \rho _{jk}(\omega ) = \frac{|S_{jk}(\omega )|^2}{S_{jj}(\omega ) S_{kk}(\omega )} \end{aligned}$$

This describes phase locking between sites based on the degree of shared phonon projection, and is related to magnitude-squared coherence and PLV in EEG analysis (Andreas et al., 2021). Notably, the Josephson-like cosine coupling in the neural Hamiltonian expectation is proportional to $\langle \cos ( \Delta \phi ) \rangle = \Re \langle e^{i \Delta \phi } \rangle$ linking it directly to the real part of the PLV and providing a physical interpretation of phase locking as energy minimisation in the coupled oscillator network.

The PAC effect arises from the amplitude-modulated phase drift term $-\chi _j r_j^2 \hat{p}_{\phi _j}$. Using a first-order expansion, $r_j^2 \approx (r_j^0)^2 + 2r_j^0 q_j(t)$, we find $\dot{\phi }_j(t) \approx \omega _j - \chi _j (r_j^0)^2 - 2\chi _j r_j^0 q_j(t)$, demonstrating a direct modulation of phase velocity by amplitude fluctuation. The strength of PAC is therefore proportional to $\chi _j r_j^0$, and the mutual information or modulation index (e.g., Tort MI) can be computed from the correlation between $q_j(t)$ and $\dot{\phi }_j(t)$ (Adriano et al., 2010; Ryan et al., 2010).

Amplitude envelopes between electrodes co-vary when those electrodes share projections onto low-frequency phonons. The AAC here is an envelope–envelope correlation

38 $$\begin{aligned} \text {Corr}(A_j, A_k) \approx \sum _{\ell =1}^{N_v} \frac{v_j^{(\ell )} v_k^{(\ell )}}{M_j M_k} \langle q_\ell ^2 \rangle \end{aligned}$$

These correlations reflect the collective structure of amplitude fluctuations across the network and complement phase-based coherence measures. They are especially informative during non-stationary or low-frequency-dominant states such as sleep and anaesthesia (Breakspear, 2017; Buzsáki, 2006).

As an illustrative application, Fig. 3 compares power spectral densities generated by the Stuart–Landau model with empirical EEG spectra recorded during eyes-open and eyes-closed conditions. Eyes-closed recordings exhibit enhanced and spectrally localized alpha-band power relative to eyes-open recordings, consistent with a transition from an exploratory to an activated dynamical regime. This qualitative correspondence is intended to demonstrate applicability of the framework rather than quantitative parameter fitting.

Figure 3a illustrates how different parameter regimes shape the spectral characteristics of neural activity. In the activated regime, sharp peaks emerge from sustained limit-cycle oscillations. The suppressed regime shows attenuated, noise-dominated activity. The exploratory state produces a flatter, broadband profile with richer high-frequency content. These results are analytically grounded in the Lorentzian form of the power spectrum, derived from the model’s linear response (see Eq. (35)).

Empirical EEG power spectra typically exhibit a broadband low-frequency background with an approximate 1/*f* dependence, upon which narrowband rhythmic peaks such as the alpha rhythm are superimposed. Within the present framework, this structure admits a natural interpretation. An isolated neural mode in effective equilibrium exhibits a mean occupation by the Bose-Einstein distribution $\langle n \rangle \approx \bar{n}$, which in the high-temperature limit scales as $\bar{n} \sim k_B T / \hbar \omega$, yielding an approximate $1/\omega$ spectral dependence. Activation through coherent drive or effective gain introduces an additional nonequilibrium contribution $\Delta n$, giving $\langle n \rangle = \bar{n} + \Delta n$. In the frequency domain, this corresponds to a finite-linewidth Lorentzian peak superimposed on a broadband 1/*f*-like background. The Stuart–Landau oscillator captures the driven coherent component $\Delta n$, while empirical EEG spectra reflect the ensemble contribution of many interacting modes and background fluctuation.

We further note the close similarity between the O1 and O2 power spectral densities across conditions, particularly in the alpha band (Fig. 3b). While no explicit cross-spectral or coherence measures are computed here, this spectral alignment is consistent with the interpretation of alpha activity as a collective mode spanning bilateral occipital regions. Within the neural phonon framework, such spatially distributed but spectrally aligned activity naturally arises from shared participation in a common oscillatory mode.

This completes the derivation of core spectral, cross-frequency, and envelope-based EEG observables. In the next section, we interpret these results in terms of cognitive states and introduce the “neural personality space” spanned by five key model parameters.

## Parameter space and the neural personality map

The operator framework derived above defines a principled mapping from model parameters to observable EEG features. We now summarise this relationship by introducing a reduced parameter space—a “neural personality map”—that captures core features of each oscillator in the network. This space is spanned by five primary quantities:

- $\lambda / \zeta$: the ratio of linear growth to nonlinear damping, which determines the equilibrium amplitude,
- $\Omega$: the eigenfrequencies of the phonon modes (obtained from the mass-normalised dynamical matrix),
- $D$: the amplitude of stochastic forcing (which sets the phonon mass $m = \hbar /2D$),
- $\chi$: the amplitude–phase coupling parameter that governs PAC strength,
- $K$: a structured matrix encoding global cortical interconnectivity; its real part shapes amplitude coherence and phonon coupling, while its spectral structure determines the richness of collective dynamics.

Together, these parameters define the intrinsic dynamics of each oscillator and the emergent structure of the neural phonon field. In practice, different brain states and modes of consciousness can be understood as regions in this five-dimensional space.

To illustrate, we list several archetypal cognitive states and map their expected parameter signatures, both at the oscillator level and in terms of phonon-level descriptors (e.g. mode occupation, coherence, entropy). See Table 2.

The “neural personality map” thus serves as a compact descriptor of system behaviour and a target space for experimental manipulation. Unlike abstract feature embeddings, the axes here derive directly from physical properties of the oscillator field. They can be modified by neuromodulation, pharmacological inputs, sensory load, or intrinsic cognitive transitions.

In the following section, we explore how these dynamics relate to topological organisation in the cortical lattice and draw parallels to quantum many-body systems (Grigory & Volovik, 2003; Gyaneshwar & Srivastava, 1990).

**Table 2:** Archetypal cognitive states in the neural personality map

State	Description	Oscillator parameters	Phonon dynamics
Suppressed	Low-frequency dominant, downregulated dynamics, sparse connectivity.	Low $\lambda /\zeta$, high *D*, low $\chi$, weak *K*	Low mode occupancy, narrowband spectrum, weak cross-mode coupling, low AAC.
Activated	Coherent, task-focused, selective rhythm dynamics.	High $\lambda /\zeta$, low *D*, high $\chi$, strong *K*	Structured $\Omega _\ell$, strong PAC, high mode coherence, narrow bandwidth.
Exploratory	High-entropy, decorrelated state with rich perceptual transitions.	Moderate $\lambda /\zeta$, high *D*, variable $\chi$, disorganised *K*	Broad $\Omega _\ell$ spectrum, many active modes, high entropy, weak coherence.
Drowsy / Transitional	Intermediate state transitioning between structured and suppressed.	Falling $\lambda /\zeta$, rising *D*, decreasing $\chi$, weakening *K*	Spectral blurring, reduced PAC and mode locking, higher noise-to-coherence ratio.
Hypercoherent / Overload	Excessive synchrony or locked dynamics.	Very low *D*, very high $\chi$, narrow $\Omega _\ell$, rigid *K*	Condensed modes, high PLV, strong PAC, low entropy, suppressed fluctuations.
Fragmented	Disintegrated dynamics with poor integration and irregular structure.	Variable $\lambda /\zeta$, moderate–high *D*, low $\chi$, fractured *K*	Scattered $\Omega _\ell$, incoherent phonon modes, weak or absent AAC.

## Topological structure and quantum parallels

The formal structure of the neural phonon Hamiltonian shares deep analogies with canonical systems in quantum condensed matter. These parallels help interpret brain state transitions, coherence, and rhythmic organisation through the lens of symmetry breaking, gauge theory, and topological excitations (Aharonov & Bohm, 1959; Grigory & Volovik, 2003; Gyaneshwar & Srivastava, 1990).

The cosine coupling term $J_{jk} \cos (\phi _j - \phi _k)$ mirrors the Josephson interaction found in superconducting qubit networks, enabling coherent phase alignment across oscillators (Barone & Paternò, 1982). In the neural setting, such coupling leads to long-range synchrony and the emergence of global modes—manifesting as PLV and cross-channel coherence in EEG. In the low-noise regime, persistent phase relationships emerge, and phase singularities (vortices) can form and self-stabilise (Desyatnikov et al., 2005).

The amplitude–phase interaction term $-\chi _j r_j^2 \hat{p}_{\phi _j}$ functions as a synthetic gauge potential, analogous to a magnetic vector potential acting on a particle confined to a ring. This term induces shifts in the phase momentum spectrum, directly modulating oscillatory frequency and enabling phenomena such as PAC. In quantum mechanics, such vector potentials are known to produce observable effects via phase shifts (e.g., Aharonov–Bohm), even in regions with zero field strength (Aharonov & Bohm, 1959).

The eigenfrequencies $\Omega _\ell$ of the phonon field define a neural excitation spectrum. Brain states with wide phonon gaps (e.g., deep sleep, anaesthesia) resemble insulating phases, whereas activated and exploratory states display a denser excitation structure akin to metallic or superfluid phases. Mode condensation, in which power becomes highly concentrated in a few low-frequency modes, corresponds to focused attention or stable oscillatory entrainment.

In spatially structured cortical networks, the phase field $\phi (\vec {r}, t)$ supports the formation of topological defects, points around which the phase winds by $2\pi$. These vortices are stable against small perturbations and have been observed in neuroimaging as rotating travelling waves, phase slips, and re-entrant loops. Their presence is a hallmark of spontaneously broken symmetry and long-range order in cortical rhythms. In modern technological analogs, such as optical vortex arrays, exciton-polariton condensates, and neuromorphic phase lattices, topologically protected phase defects are used for encoding robust information and guiding computation (Grollier et al., 2020; Lagoudakis et al., 2009; Ma et al., 2022). In neuroscience, these features may underlie cyclic transitions in cognition, working memory refresh loops, or wavefront-based processing in sensorimotor circuits (Huang et al., 2023; Ruiz et al., 2022).

Brain state transitions such as from wakefulness to sleep are interpreted as symmetry-breaking events. When the bifurcation parameter $\lambda$ crosses zero, the local energy landscape reorganises, phonon gaps shift, and the network mode structure is reconfigured. These transitions echo phase changes in many-body systems and provide a geometric way to classify and track cognitive dynamics.

Thus, the brain appears as a vibrational field with topologically structured modes. This framing not only connects neuroscience with condensed matter theory but also opens the door to using concepts like spectral invariants, topological protection, and mode entanglement to describe the architecture of thought.

## Methods, discussion and future directions

We have introduced a phonon-theoretic model of large-scale brain dynamics, rooted in the stochastic Stuart–Landau oscillator and recast via the Fokker–Planck to Schrödinger mapping into a Hamiltonian formalism. This framework enables closed-form derivation of EEG observables including power spectra, coherence, PAC, and amplitude correlations, while offering a unified physical interpretation grounded in vibrational and topological field theory (Breakspear, 2017; Gyaneshwar & Srivastava, 1990).

### Experimental EEG data and preprocessing

EEG data (Fig. 3b) were recorded in our laboratory using a 14-channel Emotiv EPOC-X headset at a sampling rate of 256 Hz during a simple resting-state protocol. The participant alternated between eyes-open and eyes-closed conditions, with each condition lasting 30 s. For the illustrative analysis presented here, two consecutive cycles of eyes-open followed by eyes-closed recordings were used.

Raw EEG signals were imported and handled using the EEGLAB toolbox (Delorme & Makeig, 2004). No additional temporal filtering, artefact rejection, or source modelling was applied prior to spatial transformation. To reduce reference dependence and mitigate volume conduction effects, the EEG signals were transformed to current source density (CSD) using the CSDtoolbox (Kayser & Tenke, 2006), yielding spatially localised estimates of cortical activity via the surface Laplacian.

The resulting CSD signals were demeaned (zero-order detrending) to remove arbitrary DC offsets introduced by the Laplacian transform. Power spectral densities were then estimated using Welch’s method with identical parameters across conditions. Spectral analysis focused on occipital electrodes (O1, O2), where alpha-band activity is known to be most prominently modulated. Mean alpha-band power was computed by integrating the power spectral density over the 8–13 Hz range. This processing pipeline was chosen to provide a clean, reference-independent illustration of eyes-open versus eyes-closed spectral differences, rather than a comprehensive EEG preprocessing or statistical analysis.

### Predictions and experimental tests

The model makes several testable predictions:

- Power spectra should show Lorentzian peaks whose centres and widths correspond to phonon mode frequencies $\Omega _\ell$ and noise-driven broadening (Mill et al., 2021).
- Phase coherence (e.g., PLV) should reflect the eigenstructure of the coupling matrix and vanish if $J_{jk}$ is set to zero (Deco et al., 2021).
- PAC strength should scale linearly with $\chi _j r_j^0$ and vanish if amplitude and phase dynamics are decoupled (Adriano et al., 2010).
- Envelope–envelope correlations should follow the overlap of eigenvectors $v_j^{(\ell )} v_k^{(\ell )}$, even when phase synchrony is absent (Buzsáki, 2006).

These predictions can be validated by projecting empirical EEG signals into the model eigenbasis and comparing predicted and observed observables under varying conditions (task, pharmacological, disease).

While the present work is theoretical, the framework is designed to interface naturally with established EEG analysis methods. The derived expressions for spectral power, phase locking, entropy, and phase–amplitude coupling align with common empirical metrics used in cognitive and clinical neuroscience, making this model amenable to future experimental validation. Full parameter identification and ensemble-level modelling are deferred to future work.

### Limitations and extensions

The present model assumes additive Gaussian noise and a harmonic approximation around the deterministic limit cycle. While analytically tractable, these assumptions neglect rare excursions, multiplicative noise, and nonlinearities at higher orders. The sources of cortical noise in EEG studies arise from many factors, ranging from the inherent quantum fluctuations of atomic motion, synaptic growth and decay, thalamic stimulus variability, ion channel stochasticity, probabilistic neurotransmitter release, glial calcium signalling, and local field interference, to broader physiological inputs such as microvascular pulsations, respiratory and cardiac entrainment, and instrumentation noise from the EEG recording setup.

Future extensions could incorporate: higher-order corrections from the full SL nonlinearity (Freyer et al., 2012), multiplicative or coloured noise terms (Risken, 1996), non-Gaussian stochastic drives (e.g., Lévy flights), coupling to neural mass or conductance-based models at the microcircuit level (Viktor et al., 2013), inclusion of subcortical or externally driven stimuli such as thalamocortical projections or sensory entrainment as dynamic forcing terms in the amplitude or phase equations.

### Conceptual implications

The formal correspondence between cortical dynamics and quantum condensed matter systems—including phase coherence, synthetic gauge potentials, excitation gaps, and topological modes—opens a novel and generative framework for both theoretical and empirical neuroscience (Aharonov & Bohm, 1959; Grigory & Volovik, 2003). Cognitive state transitions, such as those between wakefulness and sleep or focused and meditative awareness, naturally emerge as bifurcation-driven reorganisations of the phonon field. More variable or explorative modes of cognition may correspond to topologically disordered or spectrally gapless regimes, where large-scale coordination gives way to richer local diversity.

By treating brain activity as a vibrational field shaped by geometry, coupling, and stochasticity, this model moves beyond the metaphor of oscillations. It formalises brain rhythms as structured excitations with quantifiable mode architecture. In doing so, it invites a new synthesis: one in which the tools of quantum field theory, topological classification, and spectral geometry are repurposed to chart the structured evolution of brain states as revealed by EEG.

## Data Availability

The EEG data was captured from a single participant (author M.H.) using a standard eyes open - eyes closed paradigm and is available upon request.

## Appendix A: Summary of parameters and notation

**Table 3:** Summary of key symbols. Voltage in microvolts ($\mu$V), time in seconds (s)

Symbol	Meaning	Units
z	Complex state of oscillator *j*	$\mu$V
r	Amplitude (modulus)	$\mu$V
$\phi$	Phase angle	radians
$\lambda$	Linear growth rate	s$^{-1}$
$\zeta$	Nonlinear damping	$\mu$V$^{-2}\cdot$s$^{-1}$
$\omega$	Natural frequency	Hz ($\times 2\pi$)
$\chi$	Amplitude–phase coupling	$\mu$V$^{-2}\cdot$s$^{-1}$
D	Noise strength	$\mu$V$^{2}\cdot$s$^{-1}$
$\eta$	Stochastic drive	$\mu \cdot$s$^{-1/2}$
$f_r$	Amplitude forcing function	$\mu$V$\cdot$s$^{-1}$
$f_\phi$	Phase forcing function	s$^{-1}$
$K_{jk}$	Complex coupling coefficient	s$^{-1}$
$S_{jk}$	Real coupling (amplitude)	s$^{-1}$
$J_{jk}$	Phase coupling strength	s$^{-1}$
U	Drift potential	$\mu$V$^{2}\cdot$s$^{-1}$
V	Radial effective potential	$\mu$V$^{2}\cdot$s$^{-1}$
P	Probability density	$\mu$V$^{-1}$
$\psi$	Transformed wavefunction	$\mu$V$^{-1/2}$
$I_j$	Rotational inertia	s
$\Phi _{jk}$	Dynamical matrix	s$^{-2}$
$m_j$	Oscillator effective mass (temporal inertia)	s
$M_\ell$	Phonon effective mass	s
$\Omega _\ell$	Phonon eigenfrequency	Hz ($\times 2\pi$)
$q_\ell$	Mode displacement	$\mu$V
$\hat{p}_\ell$	Mode momentum operator	$\mu$V
$\hat{a}_\ell$, $\hat{a}_\ell ^\dagger$	Creation/annihilation operators	–
$\hat{n}_\ell$	Phonon occupation number	–
$\hat{p}_\phi$	Angular momentum operator	$\mu \cdot$s
$\hbar$	Fluctuation scale (EEG action)	$\mu$V$\cdot$s
